# The comparison of pure uterine serous carcinoma and mixed tumor with serous component: a single-institution review of 91 cases

**DOI:** 10.1186/s12885-023-11793-3

**Published:** 2024-01-17

**Authors:** Xuewu You, Yangyang Dong, Jiaqi Wang, Yuan Cheng, Yuanyuan Jia, Xiaobo Zhang, Jianliu Wang

**Affiliations:** 1 Department of Obstetrics and Gynecology, Peking University People’s Hospital, Beijing, 100044 P. R. China; 2 Department of Pathology, Peking University People’s Hospital, Beijing, 100044 P. R. China

**Keywords:** Uterine serous carcinoma, Mixed tumor, Histological type, Progression-free survival, Overall survival

## Abstract

**Background:**

Pure uterine serous carcinoma (p-USC) and mixed tumors with serous component (m-USC) are aggressive subtypes of endometrial cancer associated with high mortality rates. This retrospective study aimed to compare clinicopathologic features and outcomes of p-USC and m-USC in a single center.

**Methods:**

This study retrospectively reviewed patients diagnosed with USC at Peking University People’s Hospital between 2008 and 2022. T-tests and chi-square tests were used to compare clinicopathological characteristics between p-USC and m-USC. Kaplan-Meier survival curve and Cox regression analysis were used to analyze the impact of clinical and pathological variables on OS and PFS.

**Results:**

Among the 91 patients who underwent surgery, 65.9% (*n* = 60) were p-USC, and 34.1% (*n* = 31) were m-USC. Patients with p-USC had earlier menopause (*P* = 0.0217), a lower rate of progesterone receptor(PR) expression (*P* < 0.001), and were more likely to have positive peritoneal cytology (*P* = 0.0464). After a median follow-up time of 40 months, 28 (46.7%) p-USC and 9 (29%) m-USC patients had progression disease, 18 (30%) and 8 (25.8%) patients died of their disease. 5-year PFSR were 51.2% and 75.3%, respectively, and 5-year OS rates were 66% and 67.4%. Kaplan-Meier survival analysis showed that p-USC was more likely to relapse than m-USC (*P* = 0.034), but there was no significant difference in OS. Cox regression analysis showed that lymph node metastasis and surgical approach were risk factors for OS, and myoinvasion depth ≥ 1/2 was an independent risk factor for PFS.

**Conclusions:**

p-USC was more likely to relapse than m-USC, but there was no significant difference in OS between the two subtypes.

## Introduction

Uterine serous carcinoma (USC), a specific subtype of EC, accounts for less than 10% of all EC cases, but is responsible for almost 40% of EC-related deaths [1–2]. Unlike endometrioid endometrial carcinoma (EEC), USC often occurs in atrophic endometrium and is not associated with hyperestrogenism or endometrial hyperplasia [3]. Serous endometrial intraepithelial carcinoma (SEIC) is considered as the earliest form of USC [4]. Recent studies have reported that over 30% of USC patients have mixed tumors with both serous and high-grade endometrioid components [5–7]. Tumors with more than 5% serous components are referred to as mixed USC (m-USC), while tumors composed entirely of serous components are referred to as pure USC (p-USC) [8]. However, it is unclear whether the histological percentage of USC can predict the risk of recurrence or survival. It was proposed that p-USC are more likely to experience recurrence compared to those with m-USC. In contrast, others reported that patients with p-USC have the same prognosis and risk of metastasis as those with m-USC [9]. It can be argued that the etiology and pathogenesis of m-USC may differ from p-USC, possibly also resulting in a different clinical behavior [10].

Hence, the aim of this study was to assess disparities in clinicopathological characteristics between p-USC and m-USC, examine the impact of pathological subtypes on the prognosis of USC, and delve into prognostic factors across the entire USC patient population.

## Materials and methods

### Study design and patients

**Fig. 1:**
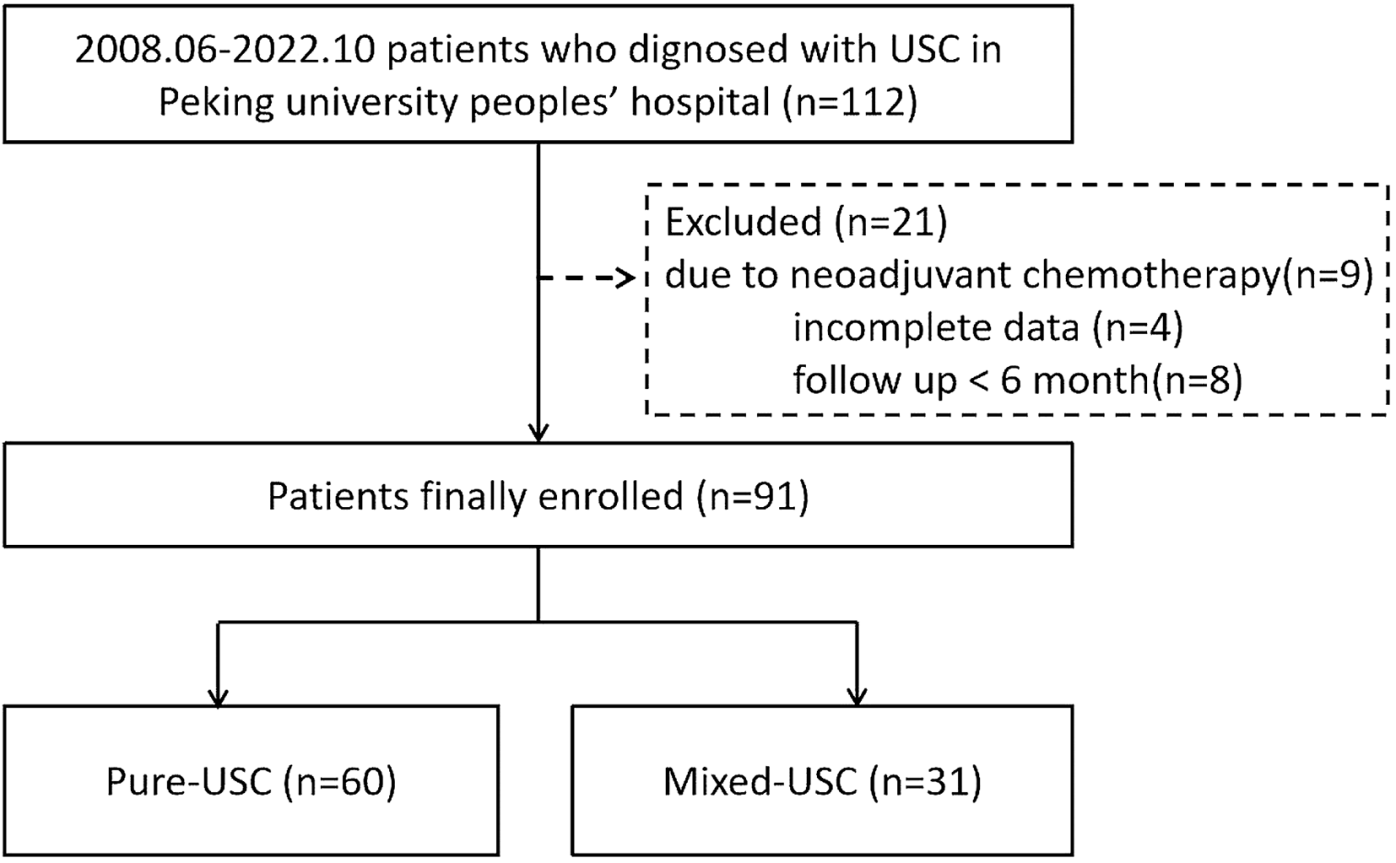
Study flow graph

**Fig. 2:**
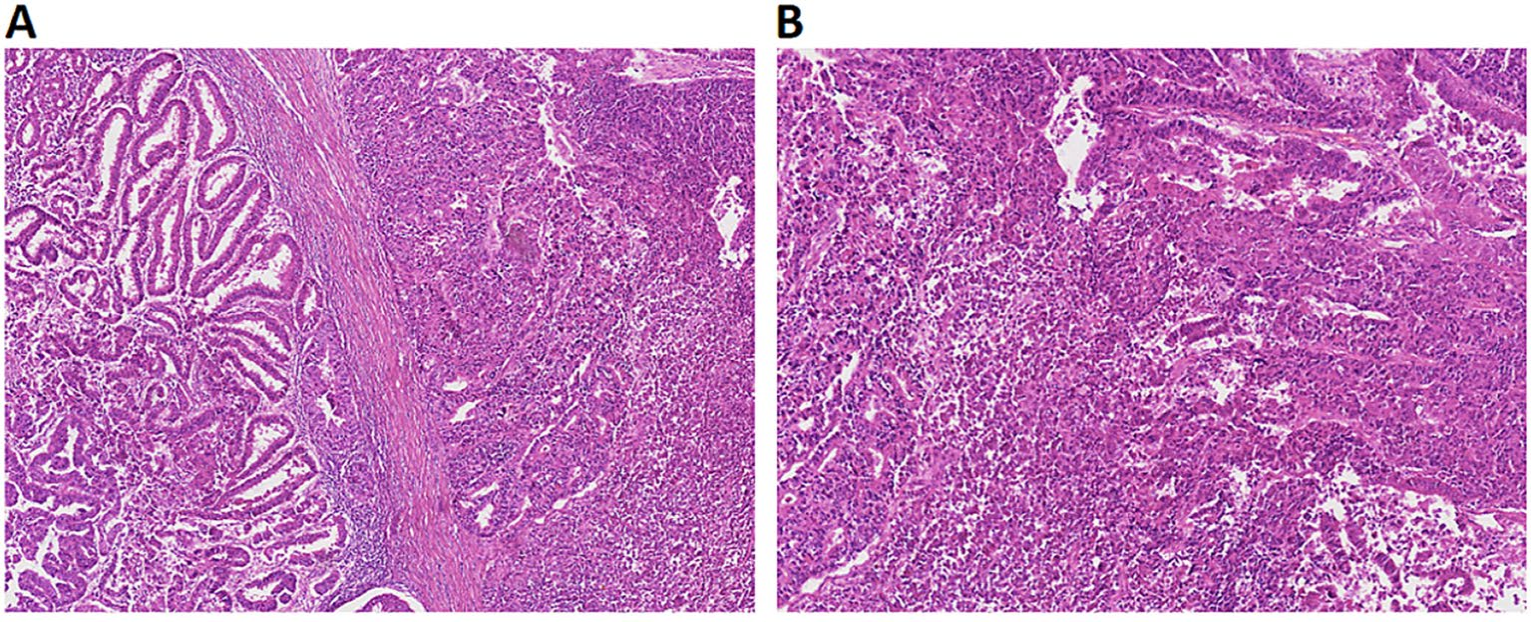
HE staining of m-USC and p-USC. m-USC (**A**) and p-USC (**B**) stained with Hematoxylin and eosin (5X)

This retrospective study included 91 women with a pathologic diagnosis of uterine serous carcinoma (USC), who received primary surgical treatment at Peking University People’s Hospital from 2008 to 2022 (Fig. 1). The study was conducted according to the guidelines of the Declaration of Helsinki and approved by the Peking University People’s Hospital Human Research and Ethics Committee (2022PHB085-001). The study collected data from clinical records and surgical pathology reports to obtain gross and histopathologic data. The inclusion criteria were patients who were pathologically diagnosed with USC after surgery, using established World Health Organization (WHO) standards. USC is characterized by a complex papillary and/or glandular structure and diffuse apparent nuclear pleomorphism. Histological examination was performed by 2 experienced gynaecological pathologists. The exclusion criteria were patients who received preoperative chemotherapy, refused surgical treatment, had incomplete clinical data, or had follow-up time less than 6 months. Patients who were completely composed of serous carcinoma components were divided into the p-USC group, while other patients with more than 5% serous component were categorized as the m-USC group (Fig. 2).

### Data collection

The study collected patient demographics and clinical characteristics, including age at diagnosis, age of menopause, parity, primary symptoms, CA-125 levels, surgery approach, adjuvant therapy, tumor personal history, tumor family history, tumor pathological characteristics (stage and pathologic factors), peritoneal cytology, and immunohistochemistry (estrogen receptor [ER], progesterone receptor [PR], and p53), for descriptive analysis. Stage was assigned according to the 2009 International Federation of Gynecology and Obstetrics (FIGO) surgical staging criteria for uterine cancer. Cases before 2009 had staging revised according to the 2009 criteria. Complete surgical staging procedure was defined as total abdominal hysterectomy, bilateral salpingo-oophorectomy, pelvic cytologic evaluations, omental biopsy, and at least pelvic lymph node sampling. Cytoreductive surgery was defined as the removal of all visible lesions to minimize the volume of residual lesions. The expression of p53, ER, and PR was considered positive when greater than 10% of the tumor cells were stained. Elevated levels of CA-125 were defined as exceeding 35 U/mL.

## Follow-up and outcomes

The patients were followed up every 3 months for the first year, every 6 months for the next 2 years, and annually thereafter until death or October 31, 2022, whichever came first. Overall survival (OS) was defined as the time (in months) from surgery to death. Progression-free survival (PFS) was defined as the time (in months) from surgery to disease progression or death. The 5-year progression-free survival rate (PFSR) was the proportion of patients alive 5 years after their primary treatment and without any signs or symptoms of USC.

### Statistical analysis

Statistical analysis was performed using SPSS 25.0, Prism 9.4.1 and R 4.2.2. Normally distributed data were expressed as mean ± standard deviation. Student’s t-test, chi-square test and Fisher exact test were used to compare the two groups. Survival analysis was conducted using the Kaplan-Meier method to assess survival time distribution, and the log-rank test was used to compare survival curves. A multivariate Cox proportional hazards model was constructed to compute hazard ratios (HRs). A p-value less than 0.05 was considered statistically significant.

## Results

### Comparison of clinical characteristics between p-USC and m-USC

A total of 91 patients who underwent surgical treatment with complete follow-up data were enrolled in the present study, including 60 (65.9%) cases of p-USC and 31 (34.1%) cases of m-USC (Fig. 1). The majority of cases in m-USC groups were serous components mixed with grade II-III EEC. As shown in Table 1, the mean age of menopause was significantly lower in p-USC compared to m-USC (50.7 ± 3.6 years vs. 52.4 ± 2.8 years, *P* = 0.0217). However, there were no statistically significant differences in age of diagnosis, parity, primary symptoms, surgical approach, FIGO stage, CA-125 levels, adjuvant treatment, tumor family history, or tumor personal history between the two groups. Among the p-USC patients, 8.3% (5/60) had a tumor personal history, of which 80% (4/5) were breast cancer. The most common primary symptoms of USC were irregular postmenopausal vaginal bleeding or vaginal drainage (78%), abdominal distension and pain (7.7%), or incidental findings in physical examination or pathological findings after hysterectomy for other diseases (14.3%).

**Table 1:** Comparison of characteristics between patients with p-USC and m-USC

Characteristics	Total-USC (*n* = 91)	Pure-USC (*n* = 60)	Mixed-USC (*n* = 31)	P-Value
Age of diagnosis	64.8 ± 9.1	65.9 ± 8.5	62.7 ± 10.0	0.0953
Age of menopause	51.3 ± 3.39	50.7 ± 3.6	52.4 ± 2.8	**0.0217**
Time from menopause to diagnosis	13.6 ± 9.4	15.2 ± 8.8	10.4 ± 9.8	0.559
Parity				0.767
<2	45(49.5%)	29 (48.3%)	16 (51.6%)	
≥ 2	46(50.5%)	31 (51.7%)	15 (48.4%)	
Symptoms				0.861
Abnormal vaginal bleeding	66(72.5%)	42 (70%)	24 (77.4%)	
Vaginal discharge	5(5.5%)	4 (6.7%)	1 (3.2%)	
Abdominal bloating	7(7.7%)	5 (8.3%)	2 (6.4%)	
others	13(14.3%)	9 (15%)	4 (12.8%)	
CA-125(U/ml)				0.644
normal	51(58.6%)	35 (60.3%)	16 (55.2%)	
elevated	36(41.4%)	23 (39.7%)	13 (44.8%)	
unknown	4	2	2	
Surgery approach				0.289
Complete staging surgery	61(83.6%)	40 (66.7%)	21 (67.7%)	
Cytoreductive surgery	12(16.4%)	10 (16.7%)	2 (6.5%)	
Others	18	10 (16.6%)	8 (25.8%)	
FIGO stage				0.174
Stage I total	42(46.2%)	23 (38.3%)	19 (61.3%)	
IA	31(34.1%)	18 (30%)	13 (41.9%)	
IB	11(12.1%)	5 (8.3%)	6 (19.4%)	
Stage II	2(2.2%)	1 (1.7%)	1 (3.2%)	
Stage III total	33(36.3%)	25 (41.7%)	8 (25.8%)	
IIIA	13(14.3%)	9 (15%)	4 (12.9%)	
IIIB	1(1.1%)	0	1 (3.2%)	
IIIC1	10(11.0%)	8 (13.3%)	2 (6.5%)	
IIIC2	9(9.9%)	8 (13.3%)	1 (3.2%)	
Stage IV total	14(15.4%)	11 (18.3)	3 (9.7%)	
IVA	5(5.5%)	3 (5%)	2 (6.5%)	
IVB	9(9.9%)	8 (13.3%)	1 (3.2%)	
Adjuvant treatment				0.504
Chemotherapy	65(84.4%)	43 (86%)	22 (81.5%)	
Chemotherapy and Radiotherapy	9(11.7%)	6 (12%)	3 (11.1%)	
None	3(3.9%)	1 (2%)	2 (7.4%)	
Unknown	14	10	4	
Tumor family history				0.787
yes	13(14.3%)	9 (15%)	4 (12.9%)	
no	78(85.7%)	51 (85%)	27 (87.1%)	
Tumor personal history				0.352
yes	6(6.6%)	5 (8.3%)	1 (3.2%)	
no	85(93.4%)	55 (91.7%)	30 (96.8%)	

**FIGO**: International Federation of Gynecology and Obstetrics; Bold values indicate significant *P*-value

### Comparison of histological characteristics between p-USC and m-USC

Table 2 displays the histological characteristics of the two groups. p-USC was more likely to be positive in peritoneal cytology compared to m-USC (37.8% vs. 15.4%, *P* = 0.046). On the other hand, m-USC was more likely to be positive in PR (67.7% vs. 23.7%, *P* < 0.001). However, there was no statistical difference in TP53 mutation rate (89.3% vs. 87.1%), SEIC (16.7% vs. 6.5%), positive rate of ER (47.5% vs. 61.3%), myometrial invasion ≥ 1/2 (46.7% vs. 32.3%), lymph-vascular space invasion(LVSI) (49.2% vs. 45.2%), or lymph node metastasis (41.8% vs. 20.7%) between the two groups. In general, among the 91 USC patients in the present study, TP53 mutation was found in 88.2% (75/85), SEIC rate was 13.2% (12/91), the positive rate of ER was 52.2% (47/90), the positive rate of PR was 38.9% (35/90), lymph node metastasis rate was 34.5% (29/84), peritoneal cytology positive rate was 29.6% (21/71), and the LVSI rate was 47.8% (43/90).

**Table 2:** Comparison of histology characteristics between patients with p-USC and m-USC

Characteristics	Total-USC (*n* = 91)	Pure-USC (*n* = 60)	Mixed-USC (*n* = 31)	P-Value
TP53-abnormity				0.759
Yes	77(88.5%)	50 (89.3%)	27 (87.1%)	
no	10(11.5%)	6 (10.7%)	4 (12.9%)	
unknown	4	4	0	
ER				0.352
positive	47(52.2%)	28 (47.5%)	19 (61.3%)	
negative	43(47.8%)	31 (52.4%)	12 (38.7%)	
unknown	1	1	0	
PR				**< 0.001**
positive	35(38.9%)	14 (23.7%)	21 (67.7%)	
negative	55(61.1%)	45 (76.3%)	10 (32.3%)	
unknown	1	1	0	
SEIC				0.172
yes	12(13.2%)	10 (16.7%)	2 (6.5%)	
no	79(86.8%)	50 (83.3%)	29 (93.5%)	
Myometrial invasion depth				0.187
<1/2	53(58.2%)	32 (53.3%)	21 (67.7%)	
≥ 1/2	38(41.8%)	28 (46.7%)	10 (32.3%)	
LVSI				0.719
Yes	43(47.8%)	29 (49.2%)	14 (45.2%)	
no	47(52.2%)	30 (50.8%)	17 (54.8%)	
unknown	1	1	0	
Lymph nodes metastasis				0.053
yes	29(34.5%)	23 (41.8%)	6 (20.7%)	
no	55(65.5%)	32 (58.2%)	23 (79.3%)	
unknown	7	5	2	
Peritoneal cytology				**0.046**
Yes	21(30.0%)	17 (37.8%)	4 (15.4%)	
no	50(70.4%)	28 (62.2%)	22 (84.6%)	
unknown	20	15	5	

**USC**: Uterine serous carcinoma; **ER**: Estrogen receptor; **PR**: Progesterone receptor; **SEIC**: Serous endometrial intraepithelial carcinoma; **LVSI**: Lymph-vascular space invasione; Bold values indicate significant *P*-value

### m-USC in relation to prognosis

**Fig. 3:**
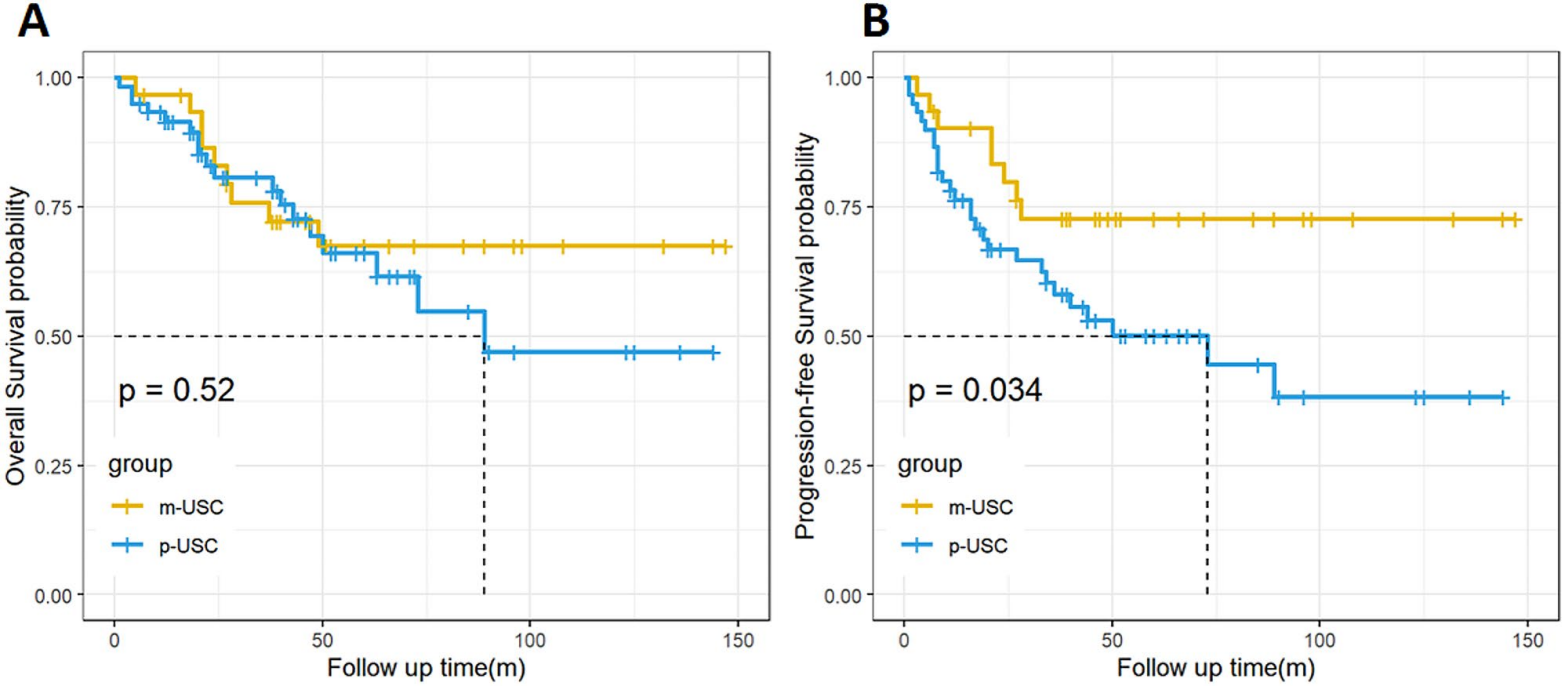
(**A,B**) OS and PFS of patients with p-USC versus m-USC. The x-axis shows the duration of follow-up (months), with OS and PFS calculated at 5 years, the y-axis shows cumulative survival from the date of diagnosis to the date of progression or death

The median follow-up time for the 91 USC patients was 40 months, with 70.3% (*n* = 64) surviving and 29.7% (*n* = 27) dying at the last follow-up. The median follow-up time for patients in the p-USC group was 39 months, with 46.7% (*n* = 28) experiencing relapse. The median PFS was 33 months, and the 5-year cumulative PFSR was 51.2%. For patients in the m-USC group, the median follow-up time was 47 months, with 29% (*n* = 9) experiencing relapse. The median PFS was 49 months, and the 5-year cumulative PFSR was 75.3% (Fig. 3). The risks of death did not differ between the two groups (*P* = 0.52), but patients in the m-USC group had lower recurrence (*P* = 0.034, Log Rank = 4.474) (Table 3**)**.

Univariate analysis showed PR, CA-125, FIGO stage, surgical approach, myometrial invasion depth, LVSI, lymph node metastasis, and peritoneal cytology were significantly associated with PFS and OS, whereas age of menopause, parity, ER and adjuvant treatment approach were not. Age over 65 years exhibited a greater likelihood of relapse (*p* = 0.046), but was not correlated with OS(Table 3). In multivariate analysis, after adjusting for factors that were significant in univariate analysis, surgery approach (hazard ratio [HR] = 2.676; 95% confidence interval (CI) = 1.061 to 6.751; *P* = 0.037) and lymph node metastasis (HR = 7.316; 95% CI = 1.0808 to 49.565; *P* = 0.041) were significantly associated with OS, while myometrial invasion depth (HR = 3.440; 95% CI = 1.146 to 10.352; *P* = 0.028) were significantly associated with PFS.

**Table 3:** Factors associated with progression-free survival and overall survival

Variables		OS Univariate analysisMultivariate analysis				PFS Univariate analysis Multivariate analysis			
		Log Rank	P-value	HR,95%CI	P-value	Log Rank	P-value	HR,95%CI	P-value
Age of diagnosis	<65, ≥ 65	2.961	0.085	--	--	3.966	**0.046**	--	--
Age of menopause	<50, ≥50	0.003	0.953	--	--	0.157	0.692	--	--
parity	<2, ≥2	0.691	0.406	--	--	0.163	0.686	--	--
ER	Positive,negative	0.731	0.393	--	--	0.148	0.701	--	--
PR	Positive,negative	3.263	0.071	--	--	8.890	**0.003**	--	--
Histological composition	Pure USC, mixed USC	0.420	0.517	--	--	4.474	**0.034**	--	--
CA-125	Normal, elveted	4.734	**0.03**	1.127 0.342–3.718	0.844	7.880	**0.005**	0.908 0.332–2.479	0.850
FIGO Stage	Early, advanced	14.618	**<0.001**	1.203 0.150–9.669	0.862	18.914	**<0.001**	1.378 0.287–6.632	0.689
Surgery approach	Staged surgery, cytoreduction surgery	17.280	**<0.001**	2.676 1.061–6.751	**0.037**	31.125	**<0.001**	1.543 0.755–3.151	0.234
Myometrial invasion depth	<1/2, ≥ 1/2	16.703	**<0.001**	2.578 0.632–10.524	0.187	16.081	**<0.001**	3.440 1.146–10.352	**0.028**
Adjuvant treatment	CT,CT and RT	0.294	0.588	--	--	1.375	0.241	--	--
LVSI	Present, absent	23.838	**<0.001**	4.814 0.787–29.431	0.089	15.766	**<0.001**	0.994 0.279–3.537	0.993
Lymph nodes metastasis	Present, absent	27.693	**<0.001**	7.316 1.080-49.565	**0.041**	29.364	**<0.001**	3.374 0.790-14.406	0.101
Peritoneal cytology	Present, absent	17.396	**<0.001**	0.737 0.193–2.822	0.656	23.514	**<0.001**	2.140 0.685–6.685	0.190

**OS**: Overall survival;**PFS**: Progression-free survival; **ER**: Estrogen receptor; **PR**: Progesterone receptor; **FIGO**: International Federation of Gynecology and Obstetrics;**LVSI**: Lymph-vascular space invasion; **CT**: chemotherapy; **RT**: Radiotherapy; Bold values indicate significant *P*-value

## Discussion

Uterine serous carcinoma(USC) is a subtype of endometrial cancer that is known to have a poor prognosis due to its aggressive nature [11]. In this study, we examined the differences between pure uterine serous carcinoma (p-USC) and mixed tumors (m-USC) and identified some key clinical and pathological features that may impact patient outcomes. Our study showed that m-USC accounted for 34.1% of all diagnosed USC, consistent with previous studies’ range of 29.5-47%. Interestingly, we observed that the age of menopause was slightly younger in p-USC patients than in m-USC patients, although there was no significant difference in the age of diagnosis. Additionally, we found that 8.2% of patients diagnosed with p-USC had a personal history of tumors, with 80% of those cases being breast cancer. In contrast, we did not observe a breast cancer history in m-USC patients. Previous studies have reported that 3.3–13% of USC patients have a history of breast cancer [5–7, 12–14], and our study supports the hypothesis that USC may be a manifestation of the hereditary breast/ovarian cancer syndrome.

Serous endometrial intraepithelial carcinoma (SEIC) is the earliest form of USC, first proposed by Sherman et al. in 1992 [15]. It is defined morphologically as replacement of endometrial surface epithelium and glands without myometrial or stromal invasion by malignant cells identical to USC tumor cells. Our study observed SEIC in both pure and mixed USC groups (20% vs. 6.9%), with up to 67% of SEIC patients having extrauterine disease, indicating the aggressive biological behavior of USC and SEIC. As a p53-driven neoplasm, p53 abnormalities exist even in SEIC lesions [16]. Our study showed that the TP53 mutation rate of p-USC and m-USC groups was 89.3% and 87.1%, respectively. In terms of copy number alternation, the most commonly amplified cancer-related genes in TP53-mutated USC were ERBB2 (16.8%), CCNE1 (16%), and MYC (12%) [17]. Following a clinical trial showing the effectiveness of anti-HER2 treatment in patients with advanced USC [18], guidelines now suggest the assessment of HER2 in advanced/recurrent USC and the addition of trastuzumab to chemotherapy.

Parity has been reported to be negatively correlated with the occurrence of endometrioid endometrial cancer (EEC) [19]. However, in our study, parity (< 1 vs. ≥ 2) was not correlated with overall survival (OS) or progression-free survival (PFS) of USC patients, and there was no difference between p-USC and m-USC groups (51.7% vs. 50%, *P* = 0.7668). Peritoneal cytology has been used as a prognostic factor of EC in many studies [20–22], but it does not affect the FIGO stage. Our study showed that the p-USC group had a higher positive peritoneal cytology rate than the m-USC group (60.7% vs. 18.2%, *P* = 0.0464). The Kaplan-Meier analysis revealed that patients with p-USC were more likely to relapse, with a median PFS of 33.5 months in p-USC and 49 months in m-USC, and the 5-year PFSR of p-USC and m-USC was 51.2% and 75.3%, respectively (Log Rank = 4.474; *P* = 0.034). However, we did not find a significant difference in OS between the two groups.

Elevated levels of CA-125 were found to be a prognostic factor for USC in the univariate analysis, but there were no significant differences in CA-125 levels between p-USC and m-USC groups. Previous studies have reported that preoperative elevated serum CA-125 levels are correlated with disease stage, extrauterine metastasis, and shortened survival in USC patients [7, 23–24]. Other histopathological factors, such as depth of myometrial invasion, lymph node involvement, and presence or absence of LVSI, have produced inconsistent results in previous USC studies [5–7]. In our study, myometrial invasion depth ≥ 1/2 and positive lymph nodes were risk factors for poor prognosis. Growdon et al. conducted a retrospective study that showed no significant difference in survival based on the presence of peritoneal cytology, LVSI, or lymph nodes status at diagnosis, and no significant difference in survival based on the type of adjuvant therapy administered [25]. However, the depth of myometrial invasion was significantly associated with survival, with a reduction in survival rate from 42.9 to 23.5% in patients with > 50% myometrial invasion (*P* = 0.027).

Multimodality therapy is typically recommended for USC due to its aggressiveness and tendency to metastasize [26]. In our study, 9.7% (3/31) of stage IA patients died, and 12.9% (4/31) relapsed, suggesting that even patients with stage IA disease may have a poor prognosis. The optimal adjuvant therapy for patients with stage IA is still controversial. A retrospective study showed that adjuvant treatment of any type (radiation therapy (RT), chemotherapy alone, or chemoradiotherapy) did not improve OS in stage IA disease [27–28]. However, other studies confirmed the benefits of adjuvant therapy in stage IA disease: adjuvant chemotherapy (with or without radiotherapy) is beneficial for reducing the recurrence rate (0–17% vs. 10%-30%) [29–31]; Platinum/paclitaxel combined radiotherapy resulted in a reduced recurrence rate compared with radiotherapy alone (7.4% vs. 20%) [29]. Although there is no consensus, the National Comprehensive Cancer Network (NCCN) recommends adjuvant chemotherapy and/or radiotherapy for patients with stage IA, except those with lesions limited to polyps [32]. Another result of our study was that chemotherapy combined with radiotherapy did not impact OS and PFS in USC patients compared with chemotherapy alone. Retrospective studies are conflicting as to whether survival improves with RT or vaginal brachytherapy (VB), likely reflecting that many cases already harbored extra-pelvic micrometastatic disease at the time of RT/VB [33–35].

Due to the rarity of this disease, few relevant studies compare the prognostic difference between p-USC and m-USC. The current study collected clinical and pathological data of USC cases admitted to our hospital during the past 14 years, which can provide reliable conclusions about the characteristics of p-USC and m-USC. We observed that patients with p-USC were more likely to relapse and had a lower 5-year PFSR than m-USC patients. These findings suggest that p-USC may have a worse prognosis than m-USC and highlight the importance of early detection and aggressive treatment for this subtype of USC.

However, our study has limitations. The proportion of serous components could not be further divided in detail due to limited availability of important information. We also failed to exhibit the expression of HER2 and other key moleculars such as PIK3CA and CCNE1, which may explain why the p-USC has a worse prognosis than the mixed group. Genetic characteristics and molecular changes of patients with p-USC and m-USC need to be further clarified. Cooperative prospective studies are necessary to better evaluate adjuvant therapies, targeted therapy, and immunotherapy for USC.

## Conclusion

Our study revealed that patients with p-USC or m-USC had similar clinical and pathological features. However, patients with p-USC exhibited a younger age of menopause, a lower positive rate of PR, and a higher likelihood of having positive peritoneal cytology. Our survival analysis indicated that p-USC was more likely to relapse than m-USC, although there was no significant difference in OS between them. As a certain subset of patients with minimal uterine disease free of serious pathologic risk factors may still experience distant metastasis, recurrence, and mortality, we recommend that both p-USC and m-USC be treated careful. Adjuvant therapy is recommended even for stage IA patients. For patients with advanced stage, optimal cytoreduction of metastatic disease followed by adjuvant platinum-based chemotherapy is the best treatment option. Levels of CA-125 may be useful in predicting advanced-stage disease. Furthermore, the use of anti-HER2 agents, anti-angiogenics, and immunotherapy combinations hold the promise of improved recurrence and survival outcomes for USC patients.

## Data Availability

All data used in this study can be obtained from the corresponding author upon reasonable request.

## Abbreviations

USC: Uterine serous carcinoma
p-USC: Pure uterine serous carcinoma
m-USC: Mixed tumors with serous component
OS: Overall survival
PFS: Progression-free survival
PR: Progesterone receptor
EEC: Endometrioid endometrial carcinoma
SEIC: Serous endometrial intraepithelial carcinoma
WHO: World Health Organization
ER: Estrogen receptor
FIGO: International Federation of Gynecology and Obstetrics
PFSR: Progression-free survival rate
HRs: Hazard ratios
CSD: Cancer-related mortality
LVSI: Lymph-vascular space invasion
RT: Radiation therapy
NCCN: National Comprehensive Cancer Network
VB: Vaginal brachytherapy
